# 2514: Governance for a decentralized informatics academic environment

**DOI:** 10.1017/cts.2017.86

**Published:** 2018-05-10

**Authors:** Thomas Fogg, Margaret Demment, Jack Chang, Kathleen Holt, Dongmei Li, Helene McMurray, David Pinto, Timothy De Ver Dye

**Affiliations:** University of Rochester Medical Center, Rochester, NY, USA

## Abstract

OBJECTIVES/SPECIFIC AIMS: Due to scope and breadth of research activity and infrastructure capacities at academic medical centers, the discipline of Biomedical Informatics is often deployed in a decentralized manner through geographically dispersed and unrelated organizational units. As a result, without a conscious strategy, an academic medical center risks redundant effort and gaps in resources, and perhaps poor coordination. A mechanism to bring together disparate organizational entities to identify, discuss, and negotiate Informatics-related concerns may produce a better institutional research environment. The University of Rochester (UR) has implemented such a strategy of Informatics governance, adapting tactics from team science, diplomacy, and deliberative engagement. METHODS/STUDY POPULATION: Based on current needs and institutional Informatics priorities, the UR’s Clinical and Translational Science Institute (CTSI) established 6 Informatics “clusters” in distinct but deliberately overlapping focal areas: (1) Data—capture, management, and analysis of all types of data for research. (2) Analytics—quantitative research across the spectrum of translational research. (3) Infrastructure—technical and computing infrastructure to support informatics. (4) Electronic health records (EHR)—(i) features within the EHR explicitly designed to address the needs of research; (ii) accessing and procuring EHR data for research. (5) Population health—Informatics design and systems expertise relevant to population health research (a key CTSI focus area). (6) Education—development, deployment, and assessment of Informatics learning opportunities for learners at all levels. Each cluster facilitates access to expertise and resources around the institution, promotes collaboration, identifies redundancy, and serves as a forum to strategize regarding institutional needs related to Biomedical Informatics. A CTSI faculty or staff member leads each cluster. To maximize effectiveness of the cluster, other members are decision-makers in the organizations they represent, or serve in a critical staff function. Clusters meet in person on a quarterly basis with more frequent electronic interaction. The clusters share documents via Box, a secure online file sharing app. The cluster coordinators meet as a group on a biweekly basis to monitor progress and make plans. RESULTS/ANTICIPATED RESULTS: There were 45 different people representing 46 distinct centers, departments or offices, and 2 outside agencies agreed to participate in the clusters. In total, 20 people represented a single organizational unit; 15 represented 2 units; 8 represented 3 units, and 2 represented 4 units. The richness and complexity of these organizational linkages illustrates the decentralized nature of Informatics at the institution and the promise of the cluster approach. DISCUSSION/SIGNIFICANCE OF IMPACT: Adapting to a decentralized Informatics environment, the CTSI established clusters that recognize and respect autonomy and capacity of a wide range of units throughout the university, creating a collaborative atmosphere for steering and implementing an overall Informatics vision. As Informatics capacity rapidly expands throughout growing biomedical research institutions without a centralized Informatics hub, this distributed, deliberative approach could offer an effective governance solution that promotes cooperation. In this model, the CTSI provides the leadership and staffing necessary to ensure progress at the institutional level around Informatics and creates a venue for communication and coordination on Informatics-related topics.

